# Unscheduled expression of CDC25B in S-phase leads to replicative stress and DNA damage

**DOI:** 10.1186/1476-4598-9-29

**Published:** 2010-02-04

**Authors:** Béatrix Bugler, Estelle Schmitt, Bernadette Aressy, Bernard Ducommun

**Affiliations:** 1 Université de Toulouse, LBCMCP, 118 route de Narbonne, F-31062 Toulouse, France; 2 CNRS, LBCMCP-UMR5088, F-31062 Toulouse, France; 3 CHU Purpan, TSA 40031, F-31059 Toulouse, France; 4 Notre Dame Hospital and Montreal Cancer Institute, Montreal H2L 4 M1, Canada

## Abstract

**Background:**

CDC25B phosphatase is a cell cycle regulator that plays a critical role in checkpoint control. Up-regulation of CDC25B expression has been documented in a variety of human cancers, however, the relationships with the alteration of the molecular mechanisms that lead to oncogenesis still remain unclear. To address this issue we have investigated, in model cell lines, the consequences of unscheduled and elevated CDC25B levels.

**Results:**

We report that increased CDC25B expression leads to DNA damage in the absence of genotoxic treatment. H2AX phosphorylation is detected in S-phase cells and requires active replication. We also report that CDC25B expression impairs DNA replication and results in an increased recruitment of the CDC45 replication factor onto chromatin. Finally, we observed chromosomal aberrations that are also enhanced upon CDC25B expression.

**Conclusion:**

Overall, our results demonstrate that a moderate and unscheduled increase in CDC25B level, as observed in a number of human tumours, is sufficient to overcome the S-phase checkpoint efficiency thus leading to replicative stress and genomic instability.

## Background

Members of the CDC25 phosphatase family (CDC25A, B and C) regulate cell cycle transitions through dephosphorylation of their substrates the CDK-Cyclin complexes. As ultimate targets of the DNA damage activated pathway, they also play a critical role in the fate of the cells in response to injury [1,2]. The currently emerging picture suggests that all three CDC25 phosphatases probably act at various stages of the cell cycle depending on the presence of the specific CDK/Cyclin complexes. Thus, CDC25B has been proposed to participate in the control of S-phase entry since specific anti-sense RNA is able to block HeLa cell replication [3] and is involved in the initiation centrosome duplication cycle in S-phase [4]. Conversely, CDC25A has been shown to play an activating role during mitosis (for review [5]).

Elevated expression of CDC25B has been documented in a growing list of human cancers [2] suggesting a potential role in the alteration of molecular processes leading to oncogenesis. The mechanisms by which the CDC25B level becomes deregulated in tumours remains unclear but it does not appear that the overexpression results from gene amplification or rearrangement. CDC25B expression can be regulated at the transcriptional [6,7], translational and post-translational levels [8,9]. During the cell cycle, CDC25B levels begin to increase from mid-S-phase, they peak during the G2-M transition and decrease in mitosis [3]. In contrast with CDC25C, CDC25B was shown to be unstable with a 30-minute half-life, its degradation being proteasome dependent [8-10]. The timing of the transition between each phase of the cell cycle must be strictly respected to maintain genomic stability. As far as CDC25B is concerned, its degradation by the proteasome pathway and/or inactivation by cytoplasmic sequestration appears to be essential to prevent activation of CDK-cyclin complexes and to avoid checkpoint overcome.

Very little is known about the mechanisms by which increased CDC25B expression contributes to the oncogenesis process. It has been shown that overexpression of CDC25B results in checkpoint bypasss and premature entry into mitosis [11,12]. We also recently reported that moderate CDC25B expression is sufficient to allow bypass of a G2/M checkpoint activated by DNA damage, thus resulting in increased sensitivity to genotoxics and increased mutagenesis [11]. Accordingly, it has been proposed that after DNA damage CDC25B accumulation [13] triggers the train of the molecular events leading to checkpoint recovery and progression in mitosis [14].

However, as mentioned above all three CDC25 phosphatases have been shown to be involved in the control of CDK-cyclin activities at the G1-S transition and in S-phase [15-17]. It is therefore tempting to speculate that in addition to critically perturbing the G2-M checkpoint, elevated and unscheduled levels of one of these phosphatases to an extent similar to that observed in human tumours might also have deleterious effects on the other key transitions.

In this study we have investigated cell cycle progression in response to unscheduled expression of CDC25B and found dramatic effects during DNA replication leading to replicative stress and genomic instability. These results emphasize the relevance of the study of its expression in human tumours and shed light on its potential role in oncogenesis.

## Results

### CDC25B unscheduled expression and progression in S-phase

**Figure 1:**
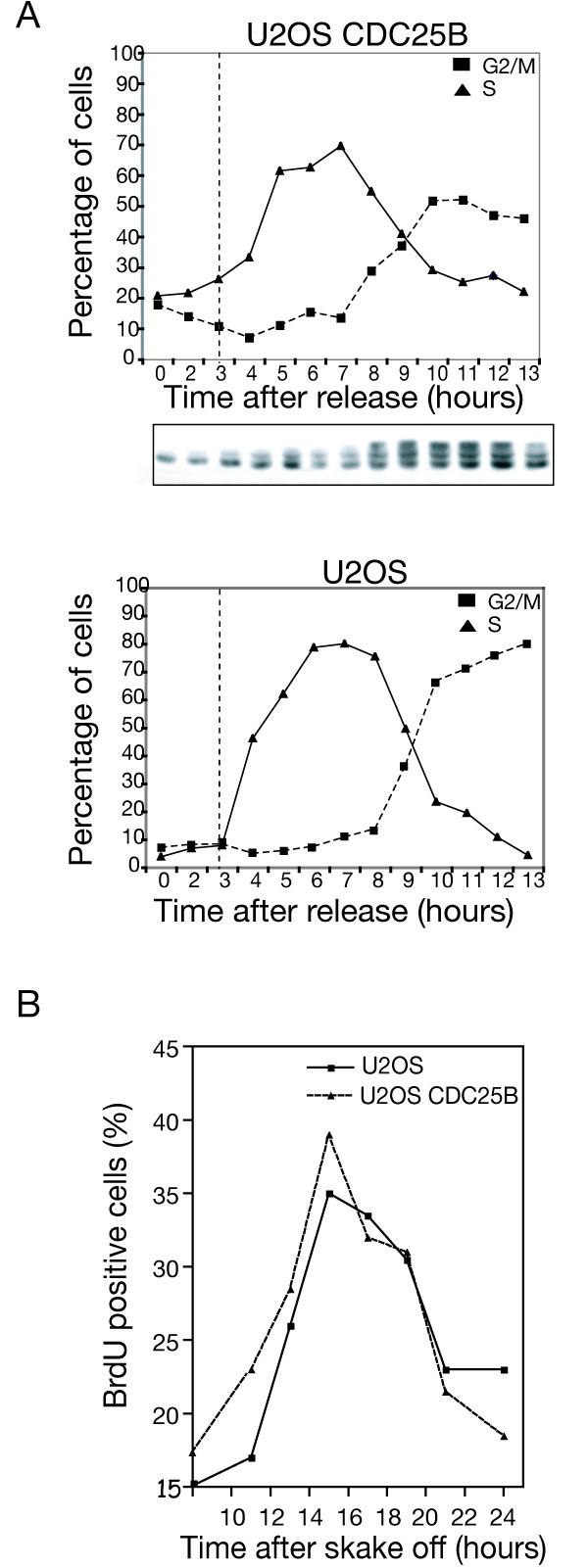
**Overexpression of CDC25B alters progression in S-phase but not replication duration**. (A) U2OS cells conditionally expressing Ha-CDC25B were synchronized by double thymidine block (Materials and Methods). At the indicated time after release, Ha-CDC25B induced cells (U2OS CDC25B) or not (U2OS) were harvested and the cell cycle distribution was monitored by flow cytometry after propidium iodide staining (PI). A western blot analysis with monoclonal anti-Ha antibodies shows the Ha-CDC25B level in the induced U2OS CDC25B cells. (B) U2OS cells conditionally expressing Ha-CDC25B were synchronized in mitosis by nocodazole treatment (100 nM, 17 h). Mitotic cells were recovered by shake off at 0 h and grown in drug free medium. Expression of CDC25B was achieved by tetracycline removal at the time of release (0 h). At indicated times after CDC25B induction, cells were labeled with BrdU (30 μM, 15 min) and the percentage of BrdU positive cells was determined by flow cytometry analysis.

To examine the impact of unscheduled CDC25B expression on cell cycle progression during S-phase we used a U2OS cell line conditionally expressing an Ha epitope-tagged CDC25B protein under the control of the tetracycline promoter [18]. We first examined cell cycle progression after synchronization by a double thymidine block and release in cells expressing Ha-CDC25B or not. Cell cycle distribution was determined by flow cytometry analyses and is shown in figure 1A as the percentage of cells in S and G2-M phase. Progression in the cell cycle appeared similar in both populations with a peak of S-phase cells at 6-7 hours. However, we noticed that an elevated level of CDC25B-expressing cells was already in S-phase immediately after thymidine block release and/or showed uncompleted DNA replication while a majority initiated the G2 phase. Similar observations were also made in cells expressing CDC25B that had been synchronized by nocodazole treatment and mitotic shake off and release with 23% of BrdU incorporation in U2OS-CDC25B cells versus 17% in U2OS cells, (Figure 1B). These observations could reflect premature entry into S-phase with subsequent perturbation of entry into mitosis as suggested by the flow cytometry analysis.

We thus examined the duration of S-phase in cells expressing Ha-CDC25B or not. These cells were BrdU labeled then chased with thymidine and collected at various times for flow cytometry analysis of BrdU positivity. Nocodazole treatment was used during the experiment to stop progression into mitosis. As shown in figure S1, Additional file 1, BrdU positivity was increased at the beginning of the U2OS-CDC25B S phase, however over time S-phase appeared identical in both cell populations indicating that S-phase duration was similar. Together with previous reports [16], these results suggest that unscheduled CDC25B expression results in a premature entry into S phase without impact on the duration of DNA replication but with possible consequences on its regulation and on its fidelity.

### Elevated CDC25B expression in S-phase induces DNA damage

**Figure 2:**
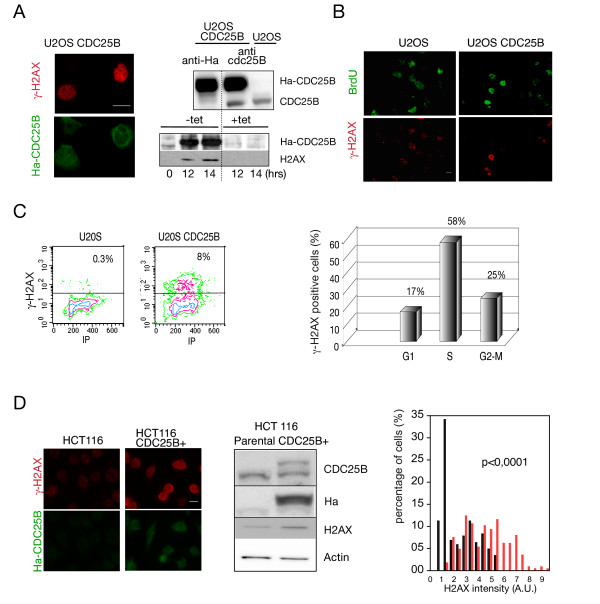
**Elevated expression of CDC25B in S-phase induces γ-H2AX labeling**. (A) Asynchronous U2OS cells overexpressing Ha-CDC25B (U2OS CDC25B) (or not) were subjected to immunofluorescence analysis after staining with anti-Ha and anti-γ-H2AX antibodies (bar = 10 μM). In the upper right part of A, a western blot analysis of Ha-CDC25B and endogenous CDC25B levels using anti-CDC25B antibodies. In the lower panel, after synchronization by nocodazole treatment and mitotic shake off followed (-tet) or not (+tet) by CDC25B induction as described in figure 1B, the cells were processed for western blot using antibodies against γ-H2AX and Ha tag (bar = 10 μM). (B) Asynchronous U2OS cells expressing Ha-CDC25B (U2OS CDC25B) or not (U2OS) were subjected to BrdU labeling for 15 min (30 μM), then processed for immunofluorescence analysis using antibodies against γ-H2AX and BrdU. (bar = 10 μM). (C) Asynchronous U2OS cells expressing Ha-CDC25B were processed for flow cytometry analysis with γ-H2AX antibodies and propidium iodide. The % indicates the quantification of γ-H2AX labeling in the global population (left panel) and in each phase of the cell cycle (right panel). Color code in flow cytometry: blue>red>green. (D) HCT116 and HCT116 CDC25B+ with a slightly elevated level of Ha-CDC25B were blocked by thymidine (2.5 mM) for 17 h then released in DMEM for 3 h. The cells were processed for immunofluorescence using antibodies against γ-H2AX and Ha-tag and for western blotting using antibodies against CDC25B, Ha-tag, γ-H2AX and actin as loading marker (bar = 10 μM). Frequency histogram from 2 immunofluorescence analyses, shows the distribution of γ-H2AX fluorescence intensity in the cells (t-test, parental HCT116 (black bars) compared to HCT116 CDC25B+ (red bars).

We next examined the possible consequences of unscheduled CDC25B expression on the occurrence of replication-linked DNA damage. With this aim, we used immunofluorescence microscopy to monitor γ-H2AX staining, a sensitive and early marker of DNA injury. As shown in figure 2A (left panel) the U2OS cells expressing Ha-CDC25B displayed a strong positive γ-H2AX staining. This positivity was also observed by western blot on total extract of cells in S-phase after synchronisation by nocodazole block and release, but was never observed in U2OS cells that do not express CDC25B (Figure 2A right panel). To examine the relationship between S-phase and the occurrence of DNA damage, we performed immunofluorescence after double staining with γ-H2AX and BrdU of U2OS cells expressing CDC25B or not. As reported in figure 2B, γ-H2AX staining was found to be largely associated with BrdU incorporating cells. Flow cytometry analysis of cell cycle distribution confirmed that while the overall percentage of cells displaying a γ-H2AX positivity was about 8% (Figure 2C, left panel), most of the U2OS-CDC25B cells displaying DNA damage were in S-phase with nearly 60% of γ-H2AX labeling in that phase of the cell cycle (right panel, Figure 2C). In contrast a very low staining level was observed in U2OS cells as shown in the scatter plots.

In order to confirm this observation in a cellular context in which the unscheduled expression of CDC25B is limited to a level frequently observed in many tumour cell lines, we made use of HCT116 cells that were engineered to stably express a moderate level of Ha-CDC25B. As shown in Figure 2D this expression is limited to about two-fold in HCT116 CDC25B+ (central panel) while in contrast a much higher expression level is achieved in U2OS cells. HCT116 and HCT116 CDC25B+ were synchronised by thymidine block and processed to immunofluorescence detection after 3 h of release. A γ-H2AX staining was observed in most of the HCT116 cells expressing Ha-CDC25B while a negligible signal was observed in the parental cell line. This observation was confirmed by the quantification of the γ-H2AX fluorescence as shown in the right panel of the figure 2D.

These observations were specific for CDC25B, as they were not observed in U2OS cells conditionally expressing CDC25C (see Figure S2, Additional file 2). Thus, our results suggest a specific role for unscheduled expression of CDC25B in the induction of DNA damage during S-phase.

### Replication is required for γ-H2AX labeling in cells expressing elevated levels of CDC25B

**Figure 3:**
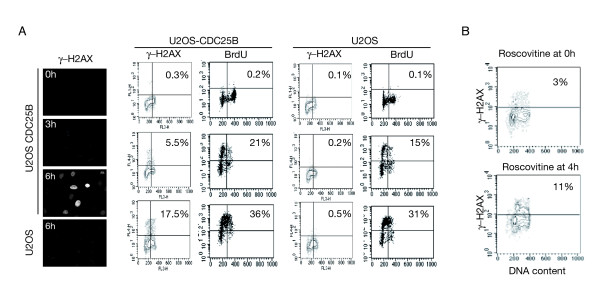
**Replication is required for γ-H2AX labeling in cells expressing elevated levels of CDC25B**. (A) U2OS cells were induced to overexpress Ha-CDC25B by tetracycline removal for 20 hours together with aphidicoline (2 μg/ml). The cells were incubated with BrdU (30 μM) for 15 min before harvesting at the indicated time (0, 3 h and 6 h) after release and staining with antibodies against γ-H2AX for immunofluorescence and rat BrdU and mouse γ-H2AX together with propidium iodide for flow cytometry analysis (bar = 10 μM). The indicated percentage corresponds to γ-H2AX and BrdU positive cells. (B) The CDK inhibitor roscovitin (10 μM) was added at the time of CDC25B induction in U2OS cells (0 h) or 4 h hours after the beginning of induction. A 17 h after CDC25B induction, flow cytometry was achieved after staining with γ-H2AX antibodies and propidium iodide. The indicated percentage corresponds to γ-H2AX positive cells.

As 60% of the cells displaying γ-H2AX staining were in S phase, we explored whether active DNA replication was necessary to observe DNA damage upon unscheduled expression of CDC25B. Asynchronous U2OS cells were induced to express Ha-CDC25B and treated at the same time with the DNA polymerase inhibitor aphidicolin to inhibit replication while increasing CDC25B expression. After 20 hours the drug was removed to resume cell cycle and the levels of γ-H2AX and BrdU incorporation were monitored by flow cytometry at each indicated time after induction of CDC25B expression. As shown in figure 3A, at the time of release from the aphidicolin block, cells were mainly arrested in G1 without BrdU incorporation and did not present any γ-H2AX positivity. By contrast, when the cell cycle was resumed by aphidicolin removal, progressive phosphorylation of γ-H2AX was clearly detected in U2OS-CDC25B by immunofluorescence staining and flow cytometry 3 and 6 hours after release, and paralleled BrdU incorporation. This positivity was not observed in the control U2OS cells population that did not expressed CDC25B. Moreover as shown in figure 3B, treatment with the CDK inhibitor roscovitin (10 μM) at the time of induction of CDC25B expression, resulted after 17 h in only 3% of γ-H2AX positivity while 11% of γ-H2AX positivity was observed when the cells were treated 4 h hours after the induction of CDC25B expression. These data suggest a correlation between the elevated level of CDC25B and its consequence on CDK2 activity, replication unwinding and γ-H2AX labeling.

DNA damage was obvious as early as 3 hours after aphidicolin block release and γ-H2AX positivity was not found to be associated with condensed, fragmented or micronucleated morphology, indicating that the DNA damage observed could not result from CDC25B-dependent mitotic catastrophe and subsequent apoptosis (see also figure S3A, Additional file 3).

Furthermore, when U2OS cells were synchronized in mitosis and released in Ha-CDC25B induction conditions, γ-H2AX labeling was detected only 13 h after synchronization when the cells entered S-phase, while Ha-CDC25B positive cells were already detected 6 hours before. Thus, despite expression of CDC25B during G1-phase, DNA damage occurred only during DNA replication and long before entry into mitosis (Figure S3B, Additional file 3).

Overall, these results indicate that DNA replication is required to observe γ-H2AX labeling upon unscheduled expression of CDC25B and strongly suggest that DNA damage is associated with replication stress and defects in the initiation and/or progression of replication forks.

### Elevated levels of CDC25B cause increased CDC45 recruitment on chromatin

**Figure 4:**
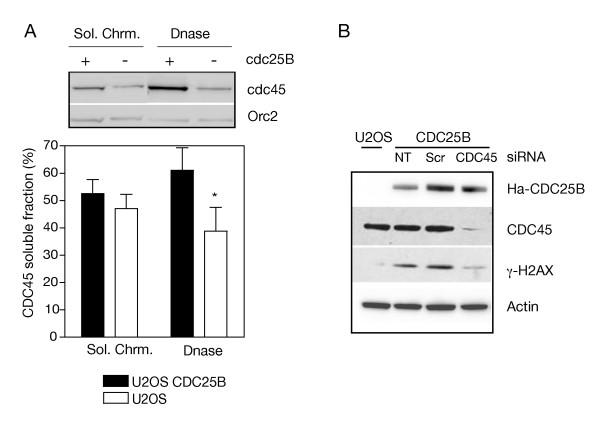
**Elevated level of CDC25B causes increased CDC45 recruitment on chromatin**. (A) U2OS cells conditionally expressing CDC25B were induced (U2OS CDC25B) or not (U2OS) in the presence of 2.5 mM thymidine for 17 h then released from the cell cycle block by thymidine removal. After 3 h, the cells were harvested and processed for western blot analysis of two chromatin extracts, the detergent-soluble fraction (Sol. Chrm.) and the DNase1 soluble fraction (DNase). The CDC45 protein level was monitored using Orc2 as loading marker. A typical western blot of one of the four independent experiments used for quantification is shown. * indicates a p value < 0.01 (t-test where U2OS CDC25B is compared to U2OS). (B) U2OS conditionally expressing CDC25B were transfected with CDC45 or control siRNA (Scr) for 28 h or not transfected (NT) then treated with 2.5 mM thymidine for 17 h in the absence of tetracycline to induce CDC25B expression. Cells were released from thymidine block by thymidine removal from the media. After 3 h, total extracts were processed for western blot analysis using anti-Ha, anti-CDC45, anti-γ-H2AX and anti-actin antibodies as loading controls. The CDC45 antibody was already tested for its specificity in [46]. Non-induced U2OS are shown as controls (U2OS).

It is well known that the initiation factor CDC45 requires the combined activation of the cyclin-dependent kinase CDK and the Dbf4-dependent kinase DDK to initiate replication firing of the inactive pre-replication complexes [19]. As CDK2-cyclinA is a bona-fide substrate for CDC25B, the likely enhanced activation of CDK2 by elevated levels of the phosphatase could result in increased phosphorylation of CDC45 resulting in the recruitment of this factor on the pre-replication complexes. To test this hypothesis, we measured the amount of CDC45 associated with the chromatin-bound fraction after DNase treatment in U2OS cells expressing elevated levels of CDC25B. The cells were harvested 3 h after release from thymidine block to enrich in S-phase cells and limit premature entry into mitosis due to CDC25B overexpression [16]. As shown in Figure 4A, the elevated and unscheduled expression of CDC25B resulted in a significant increase of chromatin-associated CDC45, whereas the CDC45 level recovered in the soluble fraction was not significantly affected. Orc2 binding was not modified by CDC25B level modulation and constitutes an internal standard. As predicted this suggests also a CDC25B involvement in the activation but not in the licensing of replication. We next examined whether DNA damage induced by unscheduled CDC25B expression was dependent on the activity of CDC45. With this aim, CDC45 expression was invalidated in U2OS cells expressing CDC25B by RNA interference and γ-H2AX was monitored by western blot. As depicted in figure 4B, DNA damage revealed by γ-H2AX labeling was significantly reduced in CDC45-depleted cells while no changes were observed in untransfected cells or in cells transfected with scrambled siRNA. Indeed, no DNA damage was detected in U2OS cells that did not express CDC25B.

These results strongly support the hypothesis that elevated and unscheduled activity of CDC25B is responsible for abnormal CDK2-cyclin activation and the subsequent phosphorylation of CDC45. This would result in the deregulation of its recruitment on the replication complexes that could likely account for the observed replication stress and the subsequent DNA damage [20-22].

### Elevated level of CDC25B impairs replication fork progression

**Figure 5:**
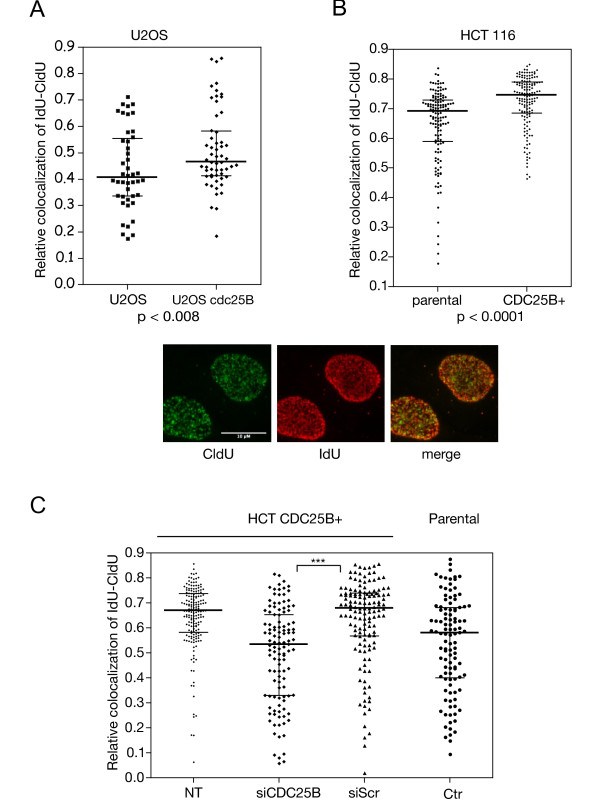
**Elevated level of CDC25B causes perturbation of S-phase progression**. (A) U2OS were induced to express Ha-CDC25B (U2OS CDC25B) or not (U2OS) then incubated in the presence of 2.5 mM thymidine for 17 h and released. Two hours after release, replication sites were pulse labeled for 30 min with CldU then for 30 min with IdU. After immunostaining with antibodies specific for CldU and IdU, the overlapping foci areas were quantified in each isolated cell and the results were expressed as the colocalization ratio (about 50 cells were analyzed). The insert shown in the central panel of this figure displays representative labeling with CldU and IdU and the typical merge figure that supports colocalization quantification using Image J software. (B) HCT116 cells expressing (CDC25B+) or not (parental) elevated levels of CDC25B (see figure 2) were processed after thymidine treatment as in (A). A total of 100 to 150 cells were analyzed. (C) HCT116 expressing elevated levels of CDC25B (HCT CDC25B+) were transfected with a CDC25B siRNA (siCDC25B) or a control siRNA (siScr) for 24 h then treated as in A and B. As controls, untransfected HCT116 CDC25B+ (NT) and parental cells (ctr). *** indicates a p value < 0.0001. A total of 130 to 170 cells were analyzed.

To gain insight into the mechanism by which unscheduled CDC25B expression could promote replication stress we examined the progression of replication forks in cells expressing or not CDC25B. With this aim, the thymidine analogs CldU and IdU were successively incorporated into DNA (see methods) and fluorescence microscopy was used to visualize, in each of the replication foci, the corresponding labeling detected with antibodies to CldU (green) and IdU (red) (Figure 5). As demonstrated by others [23], the DNA replication progression is inversely proportional to the colocalization of the two markers, the larger the overlapping areas of the CldU and IdU foci, the slower the fork migrates and vice versa. This analysis was performed in U2OS cells conditionally expressing CDC25B (Figure 5A) and in HCT116 cells expressing CDC25B (Figure 5B) that were synchronized by thymidine block and released for 2 hours to enrich the S-phase population. As shown, the relative colocalization areas of CldU-IdU were significantly more elevated in both cell types, indicating a significant perturbation of the fork progression likely due to fork stalling upon CDC25B expression.

To confirm that this observation in HCT116 CDC25B+ cells was totally dependent on CDC25B expression, we invalidated its expression by RNA interference using siRNA against CDC25B that has already been validated [11]. As presented in figure 5C, while scrambled siRNA was inefficient, the reduction of CDC25B expression with a specific siRNA led to a significant lowering of the overlapping CldU-IdU areas reflecting an increase in fork progression. These data demonstrate a clear relationship between unscheduled expression of CDC25B and deregulation of fork progression. This replicative stress is likely due to the abnormal CDC45 recruitment on replication complexes.

### Elevated levels of CDC25B cause chromosome instability

The ability of abnormal and unscheduled increased levels of CDC25B to promote replication stress resulting from a decrease of fork progression, prompted us to analyze this chromosome feature.

**Figure 6:**
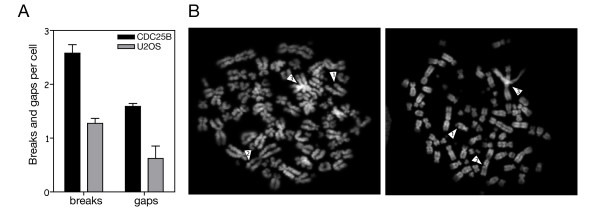
**Elevated level of CDC25B induces significant chromosomal aberrations**. (A) U2OS were induced by tetracycline removal to express Ha-CDC25B (CDC25B) or not (U2OS) during 24 h then chromosomal breaks, gaps and fusions were quantified on metaphase spreads. (B) Representative metaphase spreads with breaks (arrow 1), gaps (arrow 2) and fusions (arrow 3).

We examined chromosomal aberrations in metaphase spreads that were prepared using U2OS cells expressing CDC25B after colcemid treatment. The frequencies of chromatid and chromosome aberrations such as gaps and breaks were respectively 1.2% and 0.6% in U2OS cells whereas they rose to 2.7% and 1.6% in U2OS cells expressing CDC25B (Figure 6A). As illustrated in Figure 6B, a typical spreading of metaphase CDC25B-expressing U20S cells revealed gaps, breaks and joined chromosomes illustrating the chromosomal aberrations that were detected.

## Discussion

In this study, we show that a moderate and unscheduled increase in CDC25B protein level, comparable to the increased level that has been reported to be observed in human tumours, has a critical incidence during S phase through the generation of replication defects. We first demonstrate that abnormal level of CDC25B expression results in DNA damage essentially occurring in replicating cells. This observation is reminiscent of the premature activation of cyclin E- and cyclin A-dependent kinase observed upon CDC25A overexpression [15]. It also recalls the effect of ectopic expression of a constitutively active CDK mutant that causes DNA damage specifically in S-phase. Furthermore, chemical inhibition of CDK-cyclin can reverse the DNA damage observed in conditional Chk1 knockdown ES cells [24]. Enhanced activation of CDK2 by elevated levels of the phosphatase CDC25B has already been shown [25,26], and overexpression of CDC25B was able to overcome the unreplicated DNA checkpoint [16]. Chk1 therefore appears to be critical in controlling initiation of replication and elongation and probably acts through the modulation of CDC25 phosphatase activity [21,22,27]. One likely hypothesis to explain our observations would be that by weakening the role of Chk1, elevated and unscheduled expression of CDC25B in G1 phase would compromise the checkpoint relative to the S phase and lead to abnormal activation of CDK-cyclin activity associated to DNA replication. This effect is consistent with Chk1 haplo-insufficiency observed in some Chk1 dependent phenotypes with accumulation of DNA damage during replication and failure to restrain mitotic entry [28,29]. CDK-cyclin complexes play an essential role in regulating the activity of replication factors such as Cdc6, Cdt1 and CDC45 (reviewed in [30]) as well as in chromatin decondensation by phosphorylation of histone H1 to gain access to DNA in S phase [31]. Here we report an increased loading of the key replication factor CDC45 during S phase, upon elevated and unscheduled expression of CDC25B and a reversion of the DNA damage that was correlated to the specific depletion of CDC45. CDC45 is CDK-dependent for its activity on the chromatin and is required for origin unwinding and for the loading of the replicative polymerases [19,32]. As binding of CDC45 to chromatin is rate limiting for DNA replication, the CDC45 active form constitutes one of the critical regulator for the activation of pre-replication complexes [33] and increased loading of CDC45 in the absence of CDC25 regulation by Chk1 has already been correlated to replication stress [20-22]. Thus, an increase of CDC25B expression albeit to a minor extent close to physiological variations as observed in the HCT116 CDC25B+ cells, could phenocopy a Chk1 depletion leading to inappropriate cell cycle transition, DNA replication stress and accumulation of DNA damage.

Although S-phase duration was not changed, we also observed a decrease in the replication rate upon expression of CDC25B and we demonstrated that depletion of its expression was sufficient to rescue a normal progression. As the replication rate is inversely correlated with the density of active origins [34,35], an attractive explanation for the occurrence of DNA damage in CDC25B expressing cells would be the activation of unscheduled and unstable replication origins [36,37]. Shortening the inter origin distance induced by the formation of new active origins could increase DNA torsion stress which could in turn promote stalled and collapsed forks thus leading to double strand breaks of DNA and a slowdown of fork progression [38]. In contrast with other oncogenes CDC25B deregulation leads to replicative stress in the absence of detectable re-replication and probably through the activation of new replication origins as already observed after Myc deregulation [39].

We also report an increase in numbers of chromosomal aberrations such as gaps, breaks and joined chromosomes that illustrates the deleterious consequences of elevated CDC25B expression during S-phase and its potential role in genomic instability. In line with this observation, we previously reported that HCT116 cells, expressing elevated levels of CDC25B, displayed an elevated mutation rate compared to the parental cell line [11]. CDC25A overexpression in primary human epithelial cells was also previously shown to promote genomic instability at common fragile sites, thus accounting for the oncogenic consequences of its increased expression in human tumours [40]. In the case of CDC25B, it has been thought that as a regulator of the G2-M transition, this phosphatase did not act at the G1-S transition and in S-phase, and that the oncogenic properties associated with its overexpression in tumours could be related to G2-M checkpoint bypass and unscheduled entry into mitosis. Our findings demonstrate that this vision was incomplete. It appears that CDC25B expression must be tightly controlled and particularly in S phase, any unscheduled increase in its nuclear expression leading to replication stress and checkpoint control deficiency. Interestingly, CDC25B is mostly nuclear in G1 phase of unperturbed HeLa cells and gradually moves to the cytoplasm as cells progress to S phase depending on the presence of Cyclin B1 [16] or on the p38 mitogen activated protein kinase activation suggesting a regulation in response to various types of cellular stress [41]. Its ability to be down regulated by p53 ([42] and personal communication), well-known for its frequent inactivation in tumours, its *in vitro *transforming potential [43] and its ability to promote unscheduled entry into S-phase constitute essential features for the contribution of CDC25B to oncogenesis according to the proposed induced senescence model (for review [44]).

## Conclusion

Our findings indicate that unscheduled and moderate expression of CDC25B during S-phase is sufficient to induce replicative stress and genomic instability. Since abnormal expression of CDC25B has been found in numerous cancers (reviewed in [2,45]) our results provide new insights into the molecular mechanisms of the involvement of this phosphatase in tumorigenesis.

## Methods

### Cell culture and transfection

U2OS conditionally expressing Ha-CDC25B3 (B3 isoform) cells were grown as previously described [18]. Cells were synchronized and induced for CDC25B at the G1-S transition by a double thymidine block as follows: 16 h of treatment with 2.5 mM thymidine and 5 μg/ml tetracycline to repress the promotor, then 16 h release followed by the second thymidine block for 17 h without tetracycline to induce CDC25B. Cells were synchronized at the G2-M transition by nocodazole (100 nM, 17 h) with 5 μg/ml tetracycline then released, shaken off to retrieve mitotic cells and induced for Ha-CDC25B in the absence of tetracycline. HCT116 p53-/- clones expressing elevated levels of CDC25B were generated and grown as previously described [11].

A previously validated siRNA for CDC25B with the following sequence 5'AGACUGCAGAUACCCCUAU-3' was used. Human CDC45 siRNA pool was purchased from Santa Cruz (CA). Cells were electrotransfected using AMAXA nucleofector following the manufacturer's instructions for HCT116 and U2OS cells.

### Immunofluorescence

Mouse anti-phospho Ser139 γ-H2AX (clone JBW 301, Upstate Biotechnology, Lake Placid, NY), rabbit anti-phospho Ser139 γ-H2AX (Upstate Biotechnology), mouse anti-Ha tag (clone Ha.11 Covance), rabbit anti-phospho H3-Ser210 (Upstate Biotechnology), rat anti-BrdU (clone BU1/75 Serotec), mouse anti-BrdU (Becton Dickinson), rabbit CDC25B antibody (C-20. Santa Cruz, CA), mouse anti-actin (Chemicon, Temecula, CA), rabbit anti-CDC45 (ref. 20685. Santa Cruz). Mouse rabbit and rat anti-IgG Alexa 488 and 594 for immunofluorescence (Molecular Probes, Invitrogen), rabbit and mouse anti-HRP antibodies (Cell Signalling).

Cells cultured on glass coverslips were processed as previously described then incubated with rabbit anti-γ-H2AX and mouse anti-Ha tag or rabbit anti-phospho H3 Ser210 and mouse anti-phospho γ-H2AX followed by rabbit and mouse Alexa secondary antibody staining [12]. Cells were mounted in Vectashield anti-fade mounting medium and visualized using a DM6000 microscope (Leica, Wetzlar, Germany). For BrdU staining, cells were incubated with 30 μM BrdU (Calbiochem) for 15 min and fixed with 3.7% formaldehyde for 10 min. The cells were processed as described in [23] with some modifications: they were washed with PBS and incubated with methanol for 5 min at -20°C then treated with PBS/0.5%Triton ×100/0.02% SDS for 30 min at room temperature. DNA was denatured using freshly prepared 1.5 M HCl, then neutralized by washing with 0.1 M sodium borate (pH 8.5) and PBS. To block non-specific binding, cells were incubated in 5%PBS-BSA, 30 min to overnight at 4°C then submitted to anti-γ-H2AX or anti-BrdU for 1 h then two washes with PBS followed by mouse anti-IgG Alexa 594 and rat anti-IgG Alexa 488 respectively.

Replication focus detection with CldU and IdU was performed on U2OS or HCT116 cells blocked by thymidine (2.5 mM) for 17 h then released in DMEM for two hours. Cells were incubated in medium containing 100 μM CldU (Sigma, St Quentin, France) for 30 min then 100 μM IdU for the last 30 minutes after washing with hot medium. IdU incorporation was stopped with medium containing thymidine (1 mM) then cells were fixed with cold 70% ethanol. They were treated with 100% methanol at -20°C for 5 min, washed twice with PBS then incubated in 1.5 M HCl for 20 min. After two washes with PBS, they were incubated in 0.5% Tween20/0.25%BSA/5% fetal veal serum/PBS/(TBS) for 30 min in a humid box. Incubation in the primary antibody rat anti-BrdU against CldU and mouse anti-BrdU against IdU in TBS for 2 hours was followed by anti-rat IgG Alexa 594 and anti-mouse IgG Alexa 488 in TBS respectively. Cells were washed twice in 0.5% Tween/PBS then mounted in Vectashield solution and visualized using a DM 6000 microscope. Pictures were acquired with MetaMorph software, keeping the same intensities for each fluorescent dye for all the pictures of the same assay and the signals were measured using ImageJ software. IdU-CldU colocalization was quantified from the merge picture by dividing the colocalization area by the total area for each nucleus and the non-parametric Welch T corrected test was used to analyse the data.

### Flow cytometry

Cells were processed as previously described with mouse anti-phospho Ser139 γ-H2AX, followed by mouse anti-IgG Alexa 488 [12]. DNA was stained with propidium iodide (10 μg/ml) in the presence of RNase (5 μg/ml) and analyses were done on a FACScan flow cytometer (Cell Quest, Becton Dickinson, Mountain View, CA). For BrdU incorporation assay, the cells were incubated with 30 μM BrdU (Calbiochem) for 15 min, fixed as above then DNA was denatured by freshly prepared 1.5 M HCl, then neutralized by 0.1 M sodium borate (pH 8.5) followed by PBS. After washing in 1%PBS-BSA, rat anti-BrdU was added for 2 h together with mouse anti-phospho γ-H2AX then two PBS washes followed by rat anti-IgG Alexa 488 and mouse anti-IgG Alexa 594 staining.

### Chromatin fractionation and Western Blotting

U2OS cells were synchronized and induced for CDC25B expression at the G1-S transition by a simple thymidine block (2.5 mM, 17 h). After 3 h of thymidine release, the cells were harvested and resuspended in buffer A (10 mM HEPES pH 7.5, 1.5 mM MgCl_2_, 10 mM KCl, 10% glycerol, 0.34 M sucrose, protease inhibitors (cocktail-Complete, Roche), 10 min on ice. EDTA 10 mM was added for 30 min and this chromatin fraction obtained after centrifugation (12 000 g, 3 min, 4°C) represented the soluble fraction. The pellets were washed twice in buffer A and incubated 30 min at RT with 2000 U/ml DNaseI (Roche) and a further 30 min at 4°C with 0.5 M NaCl. The DNase solubilized chromatin fraction was obtained after centrifugation (12000 g, 3 min, 4°C).

Chromatin fractions and whole protein extracts were electrophoresed on a 4%-12% SDS gradient gel (Invitrogen, Carlsbad, CA) and analysed by Western Blotting. For protein quantification, pictures were acquired with a Bioimaging Systems, Syngene Camera and the signals measured using ImageJ software.

### Metaphase chromosomes spreads

U2OS cells were induced for CDC25B or not for 24 hrs at which point Colcemid (0.1 μg/mL; Gibco) was added for the last 3 h to accumulate mitotic cells prior to trypsinisation, centrifugation, resuspension in PBS, centrifugation and swelling in hypotonic (50 mM) KCl solution for 25 min at RT. A fixation solution of 100% ethanol/acetic acid (3:1) was added and the cells were centrifuged, rinsed twice in ethanol/acetic acid before spreading on slides and being left to dry. Chromosomes were stained with 0.05 μg/ml DAPI/PBS (Sigma) for 10 min then washed with several changes of PBS and mounted with mounting medium (Dakocytomation) prior to microscopy. About 30 spreads were scored for statistical data.

## Competing interests

The authors declare that they have no competing interests.

## Authors' contributions

BB designed, carried out the experiments and drafted the manuscript. ES performed the double block thymidine synchronisation experiment. BA constructed the HCT116 CDC25B+ cell line. BD supervised the project and finalised the manuscript. All authors have read and approved the final manuscript.

## Supplementary Material

Additional file 1 **Analysis of S phase duration**. Asynchronous cells overexpressing CDC25B (U2OS CDC25B) or not (U20S) were treated with nocodazole (200 nM) all along the assay. The cells were pulse labeled with BrdU (30 μM, 15 min) then BrdU was replaced by thymidine (1 mM). The cells were collected at the indicated times and immunostained with anti BrdU antibodies. The percentage of BrdU positive cells in S phase was determined by flow cytometry analysis. 100% correspond to the cell population before chase. As an example, the percentages of cells in S phase at 0 h and 10 h after thymidine chase were measured as shown in the two lower plots. Click here for file

Additional file 2 **Analysis of γ-H2AX staining in overexpressing CDC25C U2OS cells**. Asynchronous U2OS cells conditionally overexpressing Ha-CDC25B or Ha-CDC25C by tertracycline removal (+) [47] for 17 h were processed for flow cytometry analysis with γ-H2AX antibodies and propidium iodide. The results indicate the percentage of γ-H2AX positive cells in interphase (G1-S-G2) and mitosis. Click here for file

Additional file 3 **Analysis of γ-H2AX staining during the cell cycle**. U2OS cells conditionally expressing Ha-CDC25B were synchronized in mitosis by nocodazole treatment (100 nM, 17 h) as in figure 1B. At indicated times after CDC25B induction, cells were processed for immunofluorescence analysis using rabbit antibodies against γ-H2AX together with antibodies against phosphorylated histone H3 (panel A) or with anti-Ha to detect CDC25B (panel B). Click here for file
