# Supplementary figures and images for: Unscheduled expression of CDC25B in S-phase leads to replicative stress and DNA damage
Source: Mol Cancer. 2010 Feb 4;9:29. doi: 10.1186/1476-4598-9-29 (PMC2825247; doi:10.1186/1476-4598-9-29)

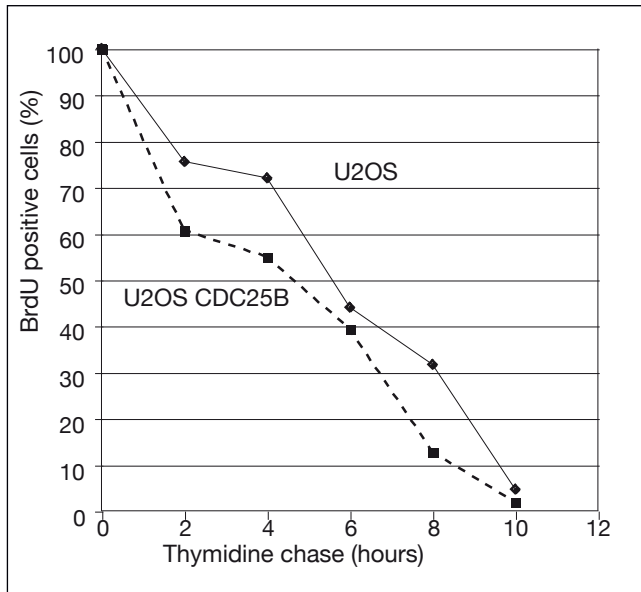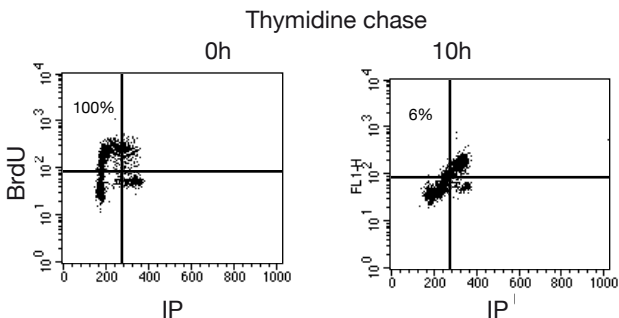

Supplement: Additional file 1 — Analysis of S phase duration. Asynchronous cells overexpressing CDC25B (U2OS CDC25B) or not (U20S) were treated with nocodazole (200 nM) all along the assay. The cells were pulse labeled with BrdU (30 μM, 15 min) then BrdU was replaced by thymidine (1 mM). The cells were collected at the indicated times and immunostained with anti BrdU antibodies. The percentage of BrdU positive cells in S phase was determined by flow cytometry analysis. 100% correspond to the cell population before chase. As an example, the percentages of cells in S phase at 0 h and 10 h after thymidine chase were measured as shown in the two lower plots. [file 1476-4598-9-29-S1.PDF]

%  $\gamma$ -H2AX

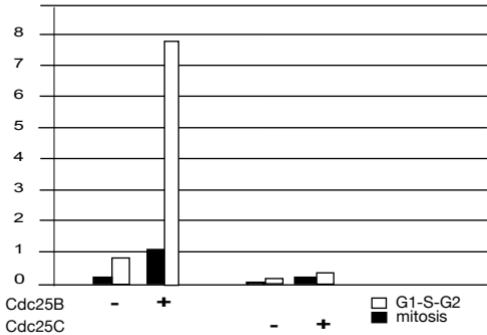

Supplement: Additional file 2 — Analysis of γ-H2AX staining in overexpressing CDC25C U2OS cells. Asynchronous U2OS cells conditionally overexpressing Ha-CDC25B or Ha-CDC25C by tertracycline removal (+) [47] for 17 h were processed for flow cytometry analysis with γ-H2AX antibodies and propidium iodide. The results indicate the percentage of γ-H2AX positive cells in interphase (G1-S-G2) and mitosis. [file 1476-4598-9-29-S2.PDF]

A

after shake off      16h      18h

H3p

$\gamma$ -H2AX

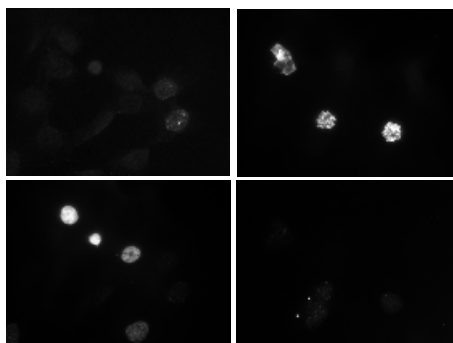

B

after shake off      4h      6h      10h

Ha-CDC25B

$\gamma$ -H2AX

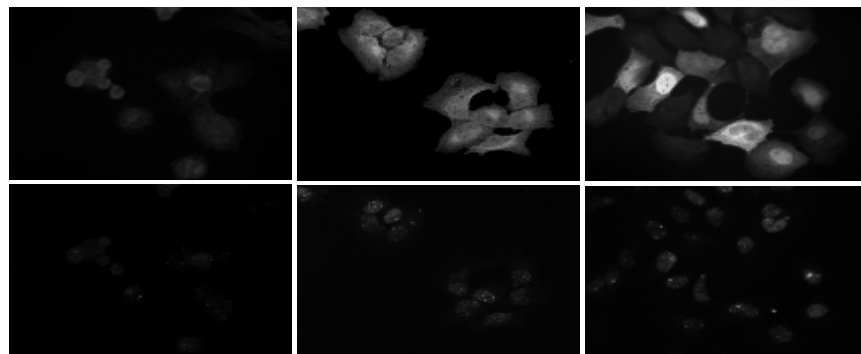

x100

12h      13h      15h      17h

Ha-CDC25B

$\gamma$ -H2AX

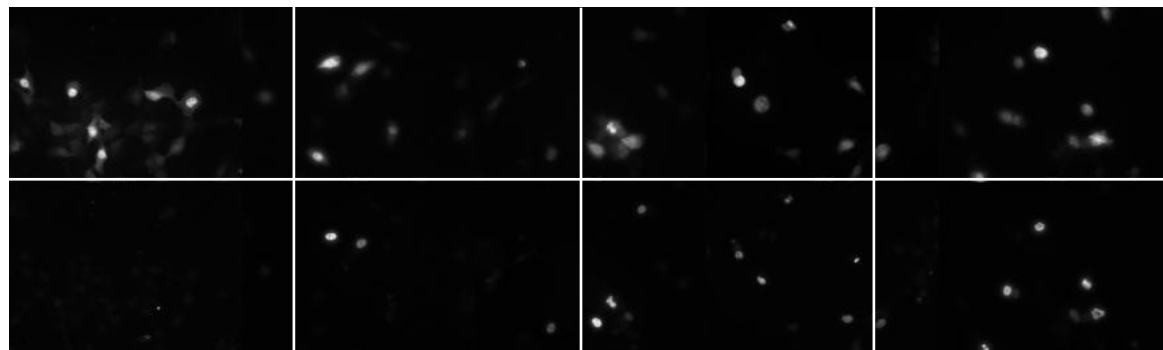

x20

Supplement: Additional file 3 — Analysis of γ-H2AX staining during the cell cycle. U2OS cells conditionally expressing Ha-CDC25B were synchronized in mitosis by nocodazole treatment (100 nM, 17 h) as in figure 1B. At indicated times after CDC25B induction, cells were processed for immunofluorescence analysis using rabbit antibodies against γ-H2AX together with antibodies against phosphorylated histone H3 (panel A) or with anti-Ha to detect CDC25B (panel B). [file 1476-4598-9-29-S3.PDF]
